# The role of Advanced Practice Nurses in creating the Kidney Transplant candidate care map (APN-preKT): a convergent-parallel mixed methods research protocol

**DOI:** 10.1186/s12912-023-01193-0

**Published:** 2023-02-17

**Authors:** Guillermo Pedreira-Robles, Victoria Morín-Fraile, Anna Bach-Pascual, Dolores Redondo-Pachón, María José Pérez-Sáez, Marta Crespo, Anna Falcó-Pegueroles, Paloma Garcimartín

**Affiliations:** 1 Nephrology Department, Hospital del Mar, Barcelona, Spain; 2 ESIMar (Mar Nursing School), Parc de Salut Mar, Universitat Pompeu Fabra affiliated, Barcelona, Spain; 3 SDHEd (Social Determinants and Health Education Research Group), IMIM (Hospital del Mar Medical Research Institute), Barcelona, Spain; 4 PhD Candidate, Nursing and Health PhD Programme, University of Barcelona, Barcelona, Spain; 5 Department of Public Health, Mental Health, and Maternal and Child Health Nursing, School of Nursing, Faculty of Medicine and Health Sciences, University of Barcelona, Barcelona, Spain; 6 Kidney Research Group (GREN), Hospital del Mar Medical Research Institute (IMIM), Barcelona, Spain; 7 Department of Fundamental Care and Medical-Surgical Nursing, School of Nursing, Faculty of Medicine and Health Sciences, University of Barcelona, Barcelona, Spain; 8 Chief Nursing Officer, Hospital del Mar, Barcelona, Spain; 9 Department of Biomedical Research in Heart Diseases, Hospital del Mar Medical Research Institute (IMIM), Barcelona, Spain

**Keywords:** Advanced Practice Nurse, Kidney transplant, Chronic Kidney Disease, Nursing, Nurse, Study protocol, Mixed Methods

## Abstract

**Background:**

Waiting time for kidney transplants (KT) is an important health determinant for patients with chronic kidney disease (CKD). During this time, ongoing evaluation and participation is necessary in order to guarantee the quality and suitability of the proposed treatment. There is no existing literature on the potential impact of inclusion of an Advanced Practice Nurse (APN) role in the hospital setting on care for CKD patients who are candidates for KT. The main objectives of this protocol are: to analyse outpatient nursing activity in the care of individuals with KT in Spain; to identify the needs of individuals who are KT candidates; and to measure the impact of the APN role through patient outcomes and experiences. These objectives are fulfilled through 5 specific related substudies.

**Methods:**

A convergent parallel mixed methods approach will be conducted between July 2021 and April 2024. Quantitative and qualitative data will be collected and analysed separately to ascertain whether the findings confirm or contradict one another. Each of the 5 substudies of the project require a specific design, sampling method, and data collection procedure in order to meet the overall objectives for the project.

**Discussion:**

The results of the project are expected to inform the design of future nursing roles and contribute to future improvements in the quality of care provided. The data that may be obtained from this protocol are limited to the specific context of the study facility and may be extrapolated but not compared to other settings due to the variability of care pathways for KT candidates internationally.

**Trial registration:**

This project was approved by the Clinical Research Ethics Committee (no.2020/9418/I). The study was supported by the “Strategic Plan for Health Research and Innovation” from the *Generalitat de Catalunya*, registration number SLT017/20/000001, with a contribution of 57,239 euros.

## Background

The prevalence of chronic kidney disease (CKD) has reached 15% among the general adult population in Spain, due primarily to increased life expectancy and comorbidities [1, 2]. Demand for specific care needs has also risen among specialist teams, who must develop appropriate, well-documented strategies for chronic disease and elderly care [3–6].

In terms of treatment for individuals with CKD, kidney transplant (KT) has been widely described as the renal replacement treatment option providing the greatest chance of survival and best quality of life, as well as the greatest cost-efficiency for the healthcare system as a whole [3, 7]. However, time from diagnosis of advanced stage CKD to KT is strongly associated with increased risk of complications and mortality among this population [8, 9]. The waiting period, which is estimated at a median of 3 years in our study setting, may be longer or shorter depending on certain characteristics of the recipient, such as blood group, age, and immunological identities in the histocompatibility system (human leukocyte antigens or HLAs) [10, 11]. In this regard, the waiting period is a determining factor for the health of individuals with CKD [12] and it comprises ongoing assessment needed to ensure the quality and suitability of the proposed treatment [13–15].

Within this context, a number of strategies have been developed to increase access to KT and prevalence of this type of treatment [16–18]. One of the strategies proposed by the Spanish National Transplant Organisation (Organización Nacional de Transplantes, ONT) is based on coordination at different structural levels (national, regional, and hospital settings) with direct involvement from interdisciplinary teams [19]. Nurses have played a major role in this process in out-of-hospital settings, contributing to the planning and management of organ donation [20]. However, in hospital settings, nurses are part of donation coordination teams, but few nurses belong to KT teams with a coordinating or care management role in the care of KT candidates [21, 22]. In addition, the international literature has identified a shortage of experienced nephrology nurses and this is of great concern to the renal community and the individuals it serves [21]. This concern stems from the population profile described above, which, as previously mentioned, requires specific care under official strategies for elderly and chronic disease care [5, 6].

Despite the limited involvement of nurses in KT, a number of studies have described the benefits that advanced practice nursing roles have brought to the care of individuals with CKD, including: improved perception of quality of life and increased satisfaction; reduced readmission rates; increased patient knowledge and greater promotion of self-care; improved patient outcomes; reduced number of face-to-face visits and travel; and reduced financial costs to the healthcare system [21, 23–27]. In addition, international guidelines on the assessment and management of KT candidates [11, 28] clearly point to the need for interdisciplinary teams, including at least one physician, one surgeon, and one nurse with expertise in the KT process, so that they can jointly assess the appropriateness of a given KT. Many of the risks described in these KT candidate assessment guidelines [11, 28] can be addressed so that the patient receives the KT in optimal conditions thanks to the role of the nurse as care manager in KT access [29–33].

To date, no studies have been found to provide sufficient data on the care needs of individuals with CKD awaiting a KT from a holistic, comprehensive perspective, nor on the presence of the APN in the assessment process in pre- and post-KT follow-up. Therefore, these issues must be analysed in an attempt to answer the research questions for this study: How many APNs are currently working in the field of KT in Spain? What are the care needs of individuals with CKD awaiting a KT? And what impact can the role of an APN have on the study population?

## Methods and analysis

### Purpose of the study

Against this backdrop, this project hypothesises that individuals with chronic kidney disease awaiting a kidney transplant have complex needs and require a care map led by an advanced practice nursing. The study aims to fulfil the following objectives:

1. To analyse outpatient nursing activity in the care of individuals with KT in Spain.
2. To identify nursing care needs in the assessment of KT candidates in order to design a standardised nursing care map.
3. To describe the care needs of patients in the process of accessing KT in terms of the number of visits, supplementary tests and economic cost.
4. To analyse the impact of the role of the APN using the patient outcomes (as measured using the Nursing Outcomes Classification or NOC) arising from the design of the standardised nursing pathway.
5. To explore the experiences of individuals with CKD in terms of their needs as KT candidates and their perceptions of the role of the APN in accessing KT.

In line with our objectives, this project will provide a detailed description of the current status of the role of the APN in access to KT and identify the practices that are in place and the interventions that could be optimised in order to improve the quality of care for this population. It will also provide an in-depth analysis of the role of the KT access nurse and the benefits that they can bring to the interdisciplinary team.

### Design

This study used a convergent parallel mixed methods design requiring the collection of quantitative and qualitative data, separate analysis, and comparison of the results to ascertain whether the findings confirmed or contradicted one another [34]. In order to address the main study objectives, 5 different substudies with interconnected approaches to the study questions were undertaken between July 2021 and April 2024.

This protocol is based on the GRAMMS (Good Reporting of A Mixed Methods Study) guidelines for high quality evidence-based methodology [35].

### Study setting and context

The study settings for this research protocol are KT access units in Spain and, more specifically, the KT Unit at the Hospital del Mar Nephrology Department in Barcelona. The results achieved after completing this study protocol may be extrapolated to contexts that are similar or not, depending on the phase of the study analyzed. Each KT unit has its care protocols determined by the specificities of its context and the population served. Despite this, the resulting data will be internationally understood and could be adapted.

To place some context in our major field of study, we are set in a tertiary level university hospital with more than 2,000 employees, 391 inpatient beds, more than 88,000 emergency visits per year, and more than 2,000 daily visits to outpatient and primary care units [36]. The Hospital del Mar Nephrology Department in Barcelona admits more than 700 patients annually and conducts more than 17,000 outpatient consultations a year. In the field of KT, a mean of 108.2 ± 13.6 procedures have been performed annually in recent years, involving an annual assessment of more than 500 individuals in KT access units, with a total of more than 180 people on average on the KT waiting list [37].

### Data collection and management

Drawing on the principles of adaptive methodology design [38]. this study protocol, which comprises different interconnected sections or substudies, can be adapted to similar settings and accommodate a wide range of modifications in order to achieve the required objectives. The substudies comprising this protocol are described below:

- Substudy 1. Outpatient nursing activity in the care of individuals with KT in Spain

A descriptive cross-sectional study with a closed sample has been designed to address objective 1. In order to ensure methodological rigour, the standards for reporting observational studies set out in the STROBE (Strengthening the Reporting of Observational studies in Epidemiology) initiative statement [39] will be obeyed.

The sample will be selected using universal sampling linked to the professionals in the 39 adult KT units in Spain (Table 1). These professionals will be administered an ad hoc questionnaire based on the literature, consisting of 18 items in a dichotomous, polytomous, or open-ended response format (Table 2). In facilities with nurses caring for individuals with CKD in the KT outpatient setting, the IDREPA instrument (*Instrumento de Definición del Rol de la Enfermera de Práctica Avanzada*, ‘Advanced Practice Nurse Role Definition Instrument’), validated in Spanish [40], will also be administered to assess the level of skills development among these specialist nurses.

**Table 1:** Activity in kidney transplantation care in Spain (ONT, 2021)

Region	Hospital		Number of adult kidney transplants per year						
			**2015**	**2016**	**2017**	**2018**	**2019**	**2020**	**2021**
Andalusia	1	H. Puerta del Mar. Cádiz	59	88	100	99	99	74	73
	2	H. Reina Sofía. Córdoba	68	53	99	82	87	69	78
	3	H. Regional. Málaga	127	175	151	176	141	106	127
	4	H. Virgen del Rocío. Sevilla	108	124	185	178	175	99	141
	5	H. Virgen de las Nieves. Granada	76	80	53	54	57	47	52
Aragón	6	H. Miguel Servet. Zaragoza	78	104	85	77	88	55	63
Asturias	7	H. Central de Asturias. Oviedo	52	57	72	77	84	82	76
Balearic Islands	8	H. Son Espases. Palma de Mallorca	52	54	70	80	83	72	66
Canary Islands	9	H. Univ. Canarias. Tenerife	74	87	65	92	79	76	73
	10	H. Insular de Gran Canaria	49	55	58	64	73	72	70
Cantabria	11	H. Marqués de Valdecilla. Santander	55	39	44	45	48	63	64
Castile-La Mancha	12	H. General. Albacete	42	54	52	45	60	26	47
	13	H. V. de la Salud. Toledo	48	41	45	37	60	19	29
Castile and León	14	Complejo Asistencial de Salamanca	62	46	54	68	64	48	57
	15	H. Clínico. Valladolid	49	63	52	80	83	51	72
Catalonia	16	H. de Bellvitge. L’Hospitalet	124	134	177	170	196	148	193
	17	H. Vall d'Hebrón. Barcelona	126	130	118	134	135	105	121
	18	H. Clínic i Provincial. Barcelona	138	159	154	150	186	139	157
	19	H. del Mar. Barcelona	84	92	116	92	124	113	96
	20	H. Germans Trías i Pujol. Badalona	73	78	89	95	105	44	69
	21	Fundació Puigvert. Barcelona	82	97	107	107	112	103	97
Valencian Community	22	H. La Fe. Valencia	93	93	133	118	116	86	115
	23	H. de Elche. Alicante	21	31	35	21	34	15	15
	24	H. d’Alacant. Alicante	102	77	80	89	72	56	52
	25	H. Dr. Peset. Valencia	60	62	78	90	70	74	84
Extremadura	26	H. Infanta Cristina. Badajoz	53	51	54	69	39	43	40
Galicia	27	H. Universitario de A Coruña	124	114	120	131	109	88	100
	28	C. H. Universitario. Santiago	44	21	40	39	67	53	40
La Rioja	29	H. San Pedro. Logroño	16	12	20	21	16	11	7
Madrid	30	H. Doce de Octubre. Madrid	130	104	103	115	117	91	103
	31	H. Ramón y Cajal. Madrid	80	58	72	74	77	77	80
	32	H. Gregorio Marañón. Madrid	41	49	41	65	65	59	52
	33	H. La Paz. Madrid	59	48	61	51	36	40	51
	34	H. Clínico San Carlos. Madrid	60	63	61	43	55	25	30
	35	H. Puerta de Hierro. Madrid	29	31	24	28	30	28	21
	36	Fundación Jiménez Díaz. Madrid	27	28	24	23	25	19	20
Murcia	37	H. Virgen de la Arrixaca. Murcia	68	70	93	82	86	69	85
Navarre	38	Clínica Univ. de Navarra. Pamplona	49	56	45	38	33	33	35
Basque Country	39	H. de Cruces. Baracaldo	153	162	170	138	161	142	142
**TOTAL**			**2835**	**2940**	**3200**	**3237**	**3347**	**2620**^**a**^	**2893**^**a**a^

^a^Decrease in total number of KTs associated with the suspension of transplant programmes due to the COVID-19 pandemic

**Table 2:** “Outpatient nursing activity in the care of individuals with KT in Spain” questionnaire

Question		Answer
1	Region	*Text*
2	Province	*Text*
3	Hospital	*Text*
4	Contact person	*Text*
5	Position of contact person	*Text*
6	Telephone	*Text*
7	Email	*Text*
8	Does your institution have dedicated kidney transplant nurses’ offices?	Yes / No
9	Level of education of the KT nurse	University Degree / Postgraduate Diploma / Master’s Degree / Doctorate
10	If so, what type?	Pre-KT / Post-KT / Living Donor / Other
11	If you consent to participate, could you please provide your contact details so that you can complete the IDREPA questionnaire (Sevilla-Guerra et al., 2018) at a mutually agreed time?	*Text*
12	In what year did the nurse start working at this office?	*Text*
13	Is this office independent from medical offices and does it perform an autonomous nursing role?	Yes / No
14	Has the person in charge of the office been assigned a specific time slot for research and teaching?	Yes / No
15	Does the person in charge of the office play a leadership and management role within the healthcare team?	Yes / No
16	How many people are seen at this office each year?	*Text*
17	The 2020 KDIGO guidelines recommend an interdisciplinary team including at least one transplant physician, one transplant surgeon, and one nurse with expertise in the psychosocial aspects of transplantation to assess and decide on patient suitability for kidney transplantation. Does your KT unit have these three professionals?	Yes / No (specify which one is missing)

Data collection will be carried out remotely. The principal investigator of the study will contact the supervising nurse as a facilitator. Access to the two questionnaires will be provided via email after they have agreed to participate and have signed the informed consent form.

We are aware that there is a possibility that the sample may be insufficient to assess statistical significance between variables. It may also be necessary to contact other facilitators or use other means of communication to recruit participants from all planned facilities. It is also possible that some administrative, ethical or legal aspects may need to be addressed at each of the facilities in order to obtain the required data. Modifications to the original protocol will be described in a final report.

- Substudy 2. Nursing care needs in the assessment of KT candidates in order to design a standardised nursing care map.

To address objective 2, a scoping review with narrative synthesis of the existing literature will be carried out in line with the PRISMA (Preferred Reporting Items for Systematic Reviews and Meta-Analyses) guidelines [41] and the five steps proposed by Arksey & O’Malley [42]. Scoping has been described as a technique for mapping the relevant literature in a particular field of interest. It tends to address broader topics in which many different study designs can be used [42, 43].

To plan the review, the research team will reach a consensus on the suitability of the descriptors, using the benchmark clinical practice guidelines for assessing KT candidates. Two researchers will conduct the review using broad, comprehensive, relevant, and freely available databases. English will be used as the search language, and articles published in English, Spanish, French, and/or Portuguese will be eligible for inclusion. Studies published in peer-reviewed healthcare journals in the past 20 years will be included to guarantee research quality. The following will be excluded: studies with paediatric patients, pilot methods, letters to the editor, study protocols without results, unavailable full-text articles, and duplicates. To synthesise the results, the findings of each included study will be summarised individually and will be grouped into consistent categories arranged by the topic describing the general idea they convey. This categorisation will enable more in-depth interpretation and a more efficient approach to addressing the review objective [44].

The results of this phase will allow us to gain a better understanding of patient needs arising from the KT access process and of the aspects that should be taken into consideration in the KT assessment process. As a result, a standardised nursing process (NP) may be developed in line with the NANDA-NIC-NOC nursing methodology [45–47] for KT candidates, which can be applied to this population on an individual basis. The NP is a systematic method structured in phases (Assessment, Diagnosis, Planning, Implementation and Evaluation) that helps nurses to provide efficient, humanised care by focusing on the attainment of outcomes agreed with the individual (Nursing Outcomes Classification, NOC) and based on the scientific process followed by professional nurses. The NP can be used to create a care plan centred on human responses that treats people holistically and views them as unique individuals requiring care that focuses specifically on them and not just on their condition [48, 49]. The resulting proposal is intended to add value to the APN’s work in this area of care.

- Substudy 3. Care needs of patients in the process of accessing KT in terms of the number of visits, supplementary tests and economic cost

A descriptive cross-sectional study with a closed sample has been designed to address objective 3. For data collection, a comprehensive review of the electronic health records of individuals assessed retrospectively will be carried out. Methodological rigour will be guaranteed through compliance with the guidelines for reporting observational studies stated in the STROBE (Strengthening the Reporting of Observational studies in Epidemiology) initiative statement [39].

The study will include the data of all individuals assessed in KT access consultations, i.e. data on the variables of interest to this study objective (Table 3). These variables were agreed upon by the members of the research team and after a thorough review of the current evidence. The cost of each activity analysed will be based on the cost tables of the referral facility. This study will report the current profile of KT candidates in a tertiary hospital in order to assess the management for this group and the direct costs that can be incurred. This analysis will allow us to propose more efficient strategies for patients and for the health system as a whole.

**Table 3:** “Care needs of patients in the process of accessing KT in terms of the number of visits, supplementary tests and economic cost” study variables

Variable Group	Variables
Sociodemographic variables	Sex; Age; Work activity; Place of birth; Place of residence
Clinical variables	Aetiology of Kidney Disease; Recurrent urine infections; Asthma; Chronic obstructive pulmonary disease; Anxiety; Depression; Heart disease; Cerebrovascular disease; Peripheral vascular disease; and other pathologies found or accompanying
Cardiovascular Risk Factors (CVRFs)	Arterial hypertension; Dyslipidaemia; Diabetes Mellitus; Body Mass Index; Sedentary lifestyle; Smoking habit
Study time and implications	Study days; Suitable for donation/transplantation; Causes that contraindicate transplantation; Renal Replacement Treatment (RRT); Estimated glomerular filtration rate of the recipient at the first visit; Estimated glomerular filtration rate of the recipient at the final visit
Visits with specialists	Number; Type; Modality and Cost of visits made as part of the assessment
Supplementary tests	Number; Type; and Cost of supplementary tests carried out

In the event that the variables of any of the study cases cannot be defined, the subjects will be excluded from the final analysis, which will mean a decrease in the size of the final sample, estimated at 500 participants based on data obtained in previous years.

- Substudy 4. Impact of the advanced practice nurses using the Nursing Outcomes Classification (NOC) arising from the design of the standardised nursing pathway

A quasi-experimental pre-post study will be carried out using a single-group. The substudy will use the standardised NP developed in the previous substudy based on the care needs of the study population and the results of the scoping review. It is assumed that care plans are sufficiently widespread in the nursing discipline, which prevents an experimental study design. The methodological rigour of this section will be based on the SQUIRE (Standards for QUality Improvement Reporting Excellence) standards for improving the quality and safety of healthcare [50].

Data will be collected for the duration of the KT candidate’s assessment, through to their inclusion in a KT programme. All patients in 2023 (estimated at 100 ± 20 subjects based on previous years’ records) will be invited to participate on a voluntary basis. The inclusion criteria will be having CKD and being under assessment for a potential KT with a minimum of three face-to-face consultations with the KT access nurse. The exclusion criterion will be declining to participate in the data collection process.

**Fig. 1:**
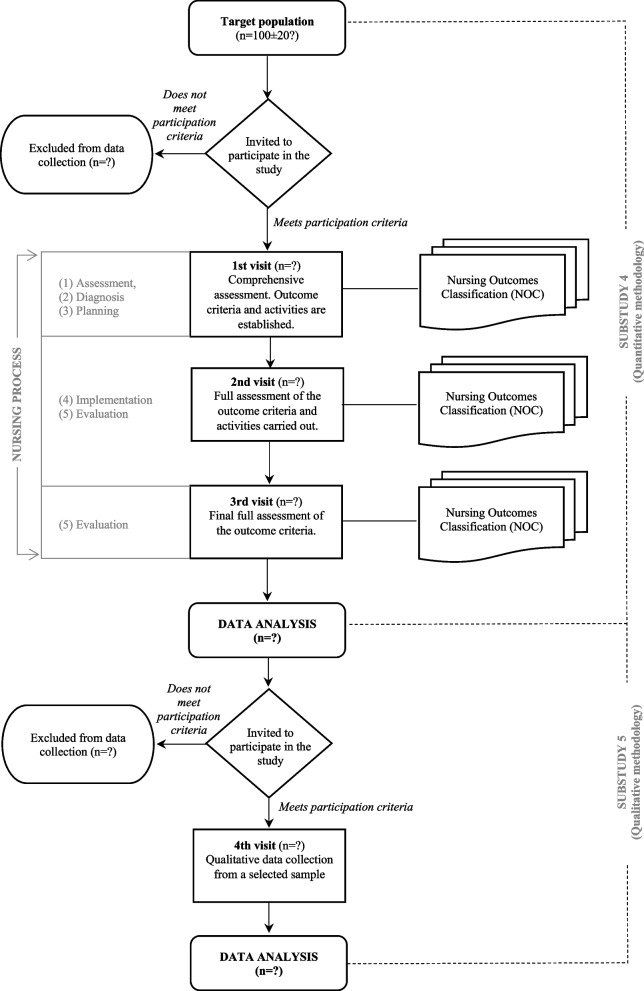
Diagram for substudies 4 and 5

Figure 1 shows the procedure for this study. At the first visit, the nurse will recruit participants (who will voluntarily sign the informed consent form), seeking to ensure follow-up and study completion based on the participant retention strategies outlined in the relevant literature [51]. Once the patient has agreed to participate, they will be fully assessed, the nursing diagnoses will be made, and the desired outcome criteria will be planned before the final visit, along with the activities to be performed in order to achieve the proposed objectives. During the subsequent visits and at the final visit, these outcome criteria will be re-evaluated and the care plan redefined accordingly. At each of the visits, data will be collected to monitor the progress made and measure the impact of the visits and of the activities specifically designed for the participant. The approximate time frame for these visits is 3 months. Visits will be planned based on schedule availability, and the follow-up time will be reflected in the final report.

There is a possibility that the desired sample size may not be reached, may be insufficient for inferential analysis, or may not be representative of the study population due to participant refusal or loss to follow-up. In these cases, this segment of the population will be excluded and, depending on the sample size and results obtained, an extension of data collection over time may be considered in order to meet the objective.

- Substudy 5. Experiences of individuals with chronic kidney disease in terms of their needs as kidney transplantation candidates and their perceptions of the advanced practice nurses’ role.

This substudy will use a qualitative, phenomenological approach, an open sampling method [52, 53], and the consolidated criteria for reporting qualitative research (COREQ) checklist [54] for methodological rigour.

The participants’ accounts will be collected in order to gain insight into their experiences of the care process as KT candidates. Participants will be selected using theoretical convenience sampling and respecting the intersectionality framework [55, 56], in order to maximise the degree of narrative variability and eliminating the bias that any factor may offer. Thus, individuals with CKD being assessed for KT will be interviewed (Fig. 1), until theoretical data saturation is reached. In line with the relevant scientific literature [57, 58] and our overall research objectives, data will be collected using semi-structured interviews based on conversational techniques. An interview script has been designed, covering the most relevant ideas or topics (salient categories) to be investigated, and ensuring clarity and appropriateness (Table 4). Once informed consent to participate has been secured, interviews will be conducted face-to-face by the principal investigator between 2023 and 2024. Participants will be able to choose the space (at the hospital or in another location) where they feel most comfortable for the interview. Interviews will always take place in a room behind closed doors in the presence of the interviewer and the interviewee only and will last approximately 40–60 min. They will be audio-recorded for subsequent transcription, and both the recording and transcription will be anonymised by assigning numerical codes. The interviewer will take field notes to record significant aspects of the interview, which will be used in the analysis. The final sample will be determined by data saturation.

**Table 4:** “Experiences of individuals with chronic kidney disease in terms of their needs as kidney transplantation candidates and their perceptions of the advanced practice nurses’ role” semi-structured interview script

**Meaning of disease and KT**	
A.	To begin with, could you tell me a little about your disease, what treatment (dialysis) you are currently undergoing, when you were diagnosed with the disease and how it has evolved since then?
B.	How have you experienced this disease process? What has it meant to you?
C.	What does kidney disease mean for your family and those around you?
D.	What does it mean to you to be able to get a transplant?
E.	What does it mean for your family and your environment that you can be transplanted?
**Needs during the KT study process **	
F.	Tell me, how did you get an appointment to go to the hospital the first day? As was?
G.	What was explained to you and your family that first day?
H.	What have you needed during the time you have made visits and tests at the Hospital?
I	Has your family needed anything?
J.	How many times have you had to come to the hospital from the first day until they told you that you were on the waiting list? Is it a lot or a little?
K.	Now that you are on the waiting list for a kidney transplant, do you need anything from the professionals who have treated you?
L.	Do you think your family needs something from the professionals?
**The KT access nurse**	
M.	Who is the nurse who has accompanied you during the time that you have gone to the hospital to assess the KT?
N.	What does this nurse do and how has it helped you?
O.	Have you ever needed to contact her? How did you do it and why?
P.	Do you think that you will ever need to contact the professionals who have treated you? Why?
Q.	How would you like to be cared for by the nurse? What is most important to you?
R.	What have you and the nurse worked on together to prepare for the transplant? What important things for you while waiting to be transplanted have you explained to the nurse?
S.	Can you imagine there being no transplant nurse in the hospital? How do you think your attention would be?
**Conclusion**	
T.	Finally, is there anything important that I have not asked you or that you would like to expand on?

Taking a qualitative approach that harnesses the voices of patients themselves, this final section of the protocol will allow us to interpret and give meaning to the results from the previous phases and to the impact of the nurse’s role on patient care. The data obtained from the project as a whole will be methodologically triangulated to facilitate its interpretation, as outlined in the relevant literature [59]. Additionally, this final study also aims to eliminate the bias introduced by other interventions occurring at the same time as the study intervention, as informants are able to discern between them as they take the lead in their own care processes [60]. Once all the substudies are finished, new research proposals may emerge. Preliminarily, we consider possible gaps in the analysis of the needs of this process such as: family members, professionals, managers, etc.

## Data analysis

### Quantitative parallel analysis

For the quantitative part of the project, the results will be recorded and analysed using the databases agreed by the research team. This process will be completed by the principal investigator using the statistical software IBM SPSS statistics version 26 (IBM Corporation) and Microsoft Excel version 15.

The IT platform at the study facility (IMASIS) will also be used, as it ensures data confidentiality by transferring data to encrypted secondary databases, preventing codes from being associated with participants.

With regard to the analysis, the study variables will be presented using a descriptive approach, showing absolute and relative frequencies for qualitative variables; means and standard deviations for quantitative variables in the case of parametric distribution of the data; and medians and interquartile ranges in the case of non-parametric distribution of the data. Multivariate analysis will be considered for each of the sections depending on the variables and results found in order to further interpret the potential resulting inferences.

### Qualitative parallel analysis

To analyse the data from the qualitative part of the project, the 7 phases proposed by Colazzi [61] will be followed using the Nvivo version 8 software. Coding into meanings and categories will be carried out by consensus among the members of the research team, who will meet on a regular basis to discuss the results obtained and the resulting analysis (researcher triangulation). This process will ensure that the criteria of credibility, transferability, dependability, and confirmability are maintained throughout the data collection and analysis process, as described in the literature [62]. The triangulation criteria will be set in line with the literature [63] and data saturation will be assessed by the members of the research team according to the established rules [64].

### Mixed methods analysis

Once the results and analysis of the parallel quantitative and qualitative phases have been obtained, the research team will integrate the results to analyse them as a whole in a discursive way. This last phase, in accordance with the parallel mixed methods methodology, will allow obtaining a deeper vision from different perspectives and, at the same time, proposing future research strategies.

## Discussion

We believe that the results of this project can make a substantial contribution to the design of the care provided in the KT setting at the international level because of the gap in the existing literature in this field. The project will also enable the creation of a nursing framework that can be replicated in other institutions nationally and internationally, advancing the nursing profession and providing valuable knowledge for nurses on caring for individuals with CKD who are candidates for KT.

Once the sub-studies are completed, their results will be published in peer-reviewed journals and presented at national and international conferences. The authors’ contributions to the different manuscripts resulting from each section of the protocol will be explicitly stated in each article. The results will also be disseminated among the broader scientific community through social media, newsletter content, conferences and patient forums, and wherever else they may be of interest. In addition, the results will be made available to anyone with a scientific interest in the methodological details of the different protocols and database organisation for reproduction, once each of the sections has been published and provided that the ethical standards described in this document and in current legislation are observed.

In accordance with the methodology presented, the main limitation to consider is that it’s not possible to assume that all the parts raised will be in agreement and this could make the mixed analysis devised difficultly. At the same time, this methodology does not allow a multicenter design, since the study's specific context provides great variability.

## Data Availability

The datasets generated during the proposed study protocol will be available from the first author (GPR) upon reasonable request.

## Abbreviations

APN: Advanced practice nursing
CKD: Chronic kidney disease
KT: Kidney transplant
NP: Nursing process
ONT: Spanish national transplant organisation [*organización nacional de transplantes*]
